# To Bleach or Not to Bleach?—The Role of Bleaching in the Clinical Workflow for the Treatment of Demarcated Opacities in Anterior Teeth

**DOI:** 10.1111/jerd.70056

**Published:** 2025-11-24

**Authors:** Linda Greenwall, Marcus Cebula, Joseph Greenwall‐Cohen, Falk Schwendicke, Susanne Effenberger

**Affiliations:** ^1^ Greenwall Dental London UK; ^2^ Clinical Research Dental‐Material Gesellschaft mbH Hamburg Germany; ^3^ Department of Conservative Dentistry, Periodontology and Digital Dentistry LMU Klinikum, LMU Munich Munich Germany

**Keywords:** bleaching, demarcated opacities, developmental defects of the enamel, MIH, OHRQoL, pediatric dentistry, resin infiltration

## Abstract

**Objective:**

To present a structured, stepwise approach for the esthetic management of demarcated anterior opacities associated with molar‐incisor hypomineralization (MIH), emphasizing the role of bleaching within a minimally invasive treatment workflow.

**Clinical Considerations:**

Discoloration of anterior teeth, particularly MIH‐related opacities, can significantly affect Psychosocial well‐being and overall health. Recommended interventions range from non‐invasive and microinvasive techniques, such as bleaching and resin infiltration, to more invasive options like composite restorations. Due to the variability in opacities, a combination of treatment modalities is often necessary. From a clinical workflow perspective, MIH‐affected anterior teeth can be categorized into basic, advanced, and complex cases, depending on their appearance and anticipated treatment needs. This clinical report demonstrates the proposed stepwise approach in five cases: two basic, two advanced, and one complex, illustrating how complexity guides treatment selection.

**Conclusion:**

A minimally invasive approach should be prioritized for managing MIH‐related anterior discolorations. Bleaching serves as an important first‐line option before considering more invasive alternatives, thereby preserving hard tooth structure‐an especially critical consideration in pediatric patients.

**Clinical Significance:**

Implementing a systematic, minimally invasive treatment protocol for MIH‐affected anterior teeth can enhance esthetic outcomes while maintaining tooth integrity, thereby improving patient confidence, identity and quality of life.

## 1 Introduction

Healthy teeth are vital for daily life. They support eating, speaking and facial aesthetics, with a confident smile being strongly linked to self‐esteem and social success. In contrast, individuals with visible enamel defects often feel self‐conscious, avoid smiling, and withdraw socially, which can harm their confidence and life opportunities. This impact is especially significant for children and adolescents as they develop their identity and social connections [1, 2].

Molar‐incisor hypomineralization (MIH) is a common developmental enamel defect that negatively affects both function and aesthetics. Typically, the first permanent molars and often incisors are affected, exhibiting disrupted enamel mineralization and morphological alterations [3]. Affected enamel has reduced mineral density, fracture resistance, and hardness, along with increased porosity and protein content. Clinically, these changes present as well‐demarcated enamel opacities ranging from white‐creamy to yellow–brown, varying in size and location [4].

With a global prevalence of roughly 15.5%, including an estimated 6.9% of the population with affected incisors in addition to first permanent molars [5], MIH represents a notable public‐health issue. It significantly reduces oral health‐related quality of life (OHRQoL), with affected children being about 17–25 times more likely to experience negative impacts [6, 7, 8]. Esthetic treatment, particularly of anterior opacities, has shown good clinical outcomes and substantial OHRQoL improvement [9, 10, 11].

Several minimally invasive treatment options exist for anterior MIH lesions, including bleaching, microabrasion, resin infiltration and composite restorations [12]. Among these, bleaching is the least invasive option. It effectively lightens discolorations and renders white opacities less visible by whitening the surrounding enamel [13]. This is achieved by peroxide‐based oxidation of organic chromophores responsible for the discoloration. Adverse effects are usually mild and transient, and current evidence supports bleaching as a safe and effective option for children and adolescents [14, 15].

A more invasive measure is microabrasion, which has been shown to reduce surface discolorations [16] by removing a thin layer of surface enamel (100–200 μm) through the use of acid abrasive pastes. This has the added effect of opening the porous body of lesions to either enable access of remineralizing agents, such as CPP‐ACP [17], or to assist with resin infiltration treatment [16, 18, 19].

Resin infiltration is a microinvasive option that involves the penetration of a low‐viscosity resin into the porous structure of hypomineralized enamel, effectively stabilizing and masking the lesion. This masking effect results from the resin's refractive index closely matching that of natural enamel. For successful infiltration, the lesion body must first be made accessible, which is typically achieved by using 15%–20% hydrochloric acid to remove the surface layer—an effect that is augmented through microabrasion pre‐treatment as indicated above [20]. Although the predictability of resin infiltration has historically been limited for MIH, recent advances—especially transillumination guidance and combination protocols incorporating prior bleaching—have greatly improved success rates and patient‐reported outcomes, including socio‐emotional wellbeing [9, 10, 11, 18, 19, 21, 22, 23, 24, 25, 26, 27, 28].

Finally, composite restorations may be necessary when opacities persist or enamel loss is extensive, for example, in the case of enamel breakdown or when an extensive amount of enamel has been removed by microinvasive treatments, which might be the case for deep lesions or persistent discolorations [10, 16, 29, 30, 31].

The purpose of this report is to illustrate how to develop individualized, stepwise treatment protocols for the esthetic improvement of demarcated anterior opacities, with particular emphasis on the role of bleaching within the overall clinical workflow.

## 2 Clinical Workflow

**FIGURE 1:**
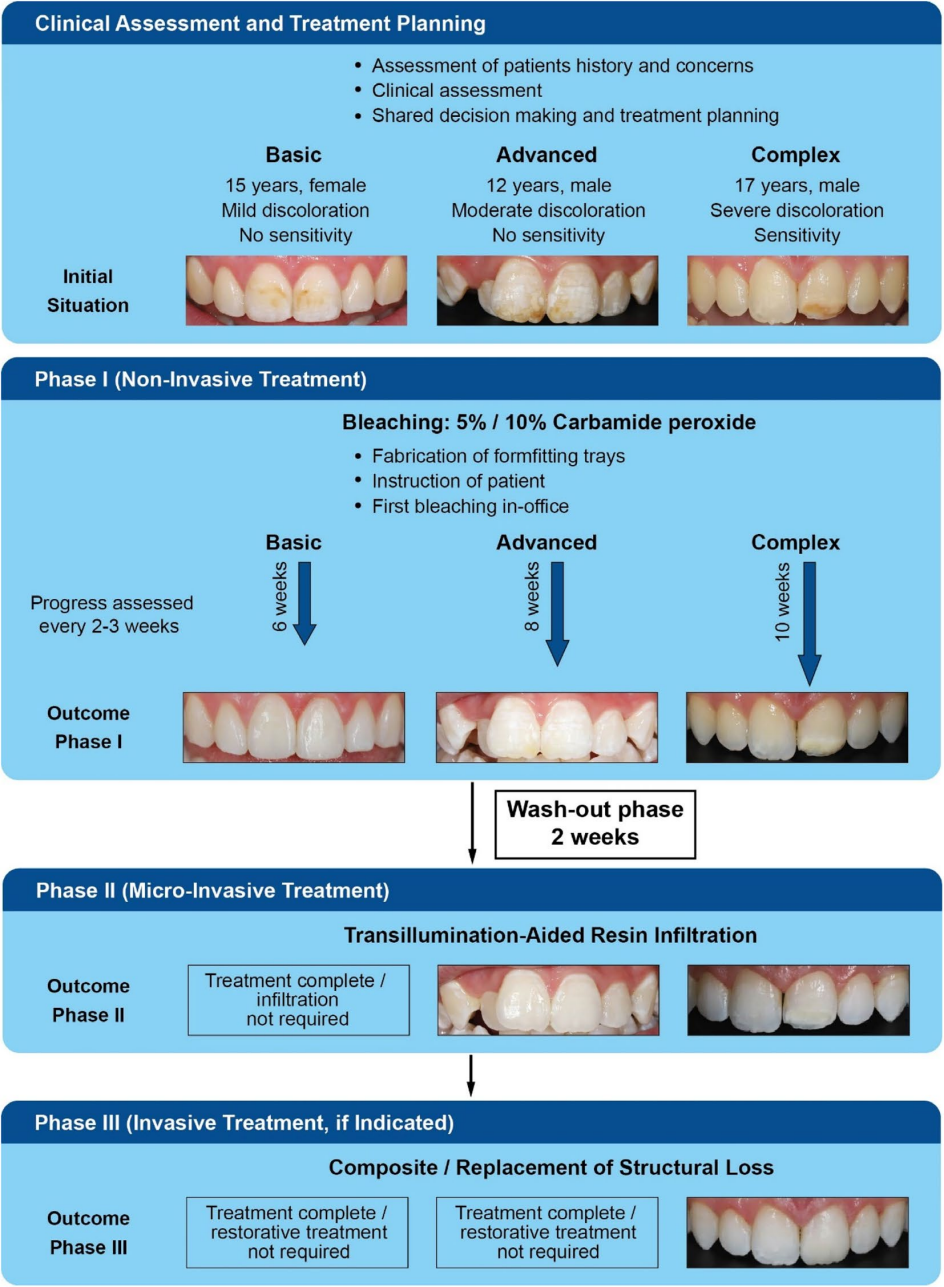
Graphic representation of the clinical workflow. Three cases—basic, advanced, and complex—are presented to illustrate the principal workflow, including clinical presentation at baseline and after each treatment phase. Sensitivity management is outlined separately in Figure 2.

Esthetic concerns affecting the OHRQoL are the primary motivation for patients seeking treatment for anterior teeth affected by demarcated opacities [9]. While various treatment options are available, the appropriate choice depends on the clinical presentation, lesion severity, psychological impact, expected outcomes, and patient age. Often, a tailored combination of treatments yields the best results [10, 11, 30, 31]. Whenever possible, treatment should remain conservative, that is, starting with the least invasive option before considering more invasive approaches, to preserve hard tooth structure, which is especially important in children [12]. Given the variety in clinical presentation and anticipated treatment requirements, MIH affected anterior teeth can be categorized into basic, advanced and complex cases within a workflow‐based classification (Figure 1, Table 1). Importantly, any treatment should only begin once the permanent teeth have fully erupted. This categorization is based on the diagnostic criteria and severity‐based management proposed by the European Academy of Paediatric Dentistry (EAPD) [12].

**TABLE 1:** Categorization of MIH cases from a workflow perspective.

	Basic	Advanced	Complex
Clinical situation	Mild‐to‐moderate MIH Mild white marks, flecks	Moderate MIH More extensive white lesions, slight discolorations, multiple lesions on many teeth	Moderate to severe MIH Complex discoloration, with multiple different shades, lesions are often very deep
Bleaching treatment^a^	*Home bleaching: 5%/10% carbamide peroxide*		
	Ca. 4–6 weeks	Ca. 6–8 weeks	Ca. 10–12 weeks
Subsequent treatment	No additional restorative dentistry required	Some microinvasive treatment (e.g., resin infiltration, microabrasion) might be needed	Complex restorative dentistry required (e.g., microinvasive treatment followed by composite restoration, veneers)

^a^ Approximate duration of bleaching treatment to achieve B1 shade (lightest shade on the Vita Classic shade guide).

To achieve successful outcomes, clear and empathic communication is vital. Patients (and parents) need realistic explanations of each modality, its limitations, and the likely esthetic improvements so expectations align with achievable outcomes. Additionally, comprehensive photographic documentation—before, during and after therapy—is strongly recommended to aid decision‐making, monitor progress, and document results. The next sections outline step‐by‐step the clinical workflow for managing demarcated opacities of varying severity.

### 2.1 Clinical Assessment and Treatment Planning

The initial step involves a thorough diagnostic evaluation and clinical assessment of the opacities to inform appropriate treatment planning. Several key factors should be considered:

- *Patient level*: Age, medical history, the ability to cooperate, psychological impact of dental appearance, and access to specialist dental care.
- *Oral level*: Number of affected teeth, DMFT, overall oral health and oral hygiene, as well as the developmental stage of the dentition and the patient's growth and eruption status.
- *Tooth level*: Color, size, and depth of the defect; presence or absence of dentin hypersensitivity or post‐eruptive enamel breakdown; and other defects such as trauma or caries.

In addition, personalized oral hygiene instructions should be provided, as patients with discolorations, particularly brown stains, often neglect oral care due to dissatisfaction with their teeth's appearance, which can further worsen the issue and compromise long‐term treatment success [32, 33].

### 2.2 Phase I—Noninvasive Treatment (Bleaching)

**FIGURE 2:**
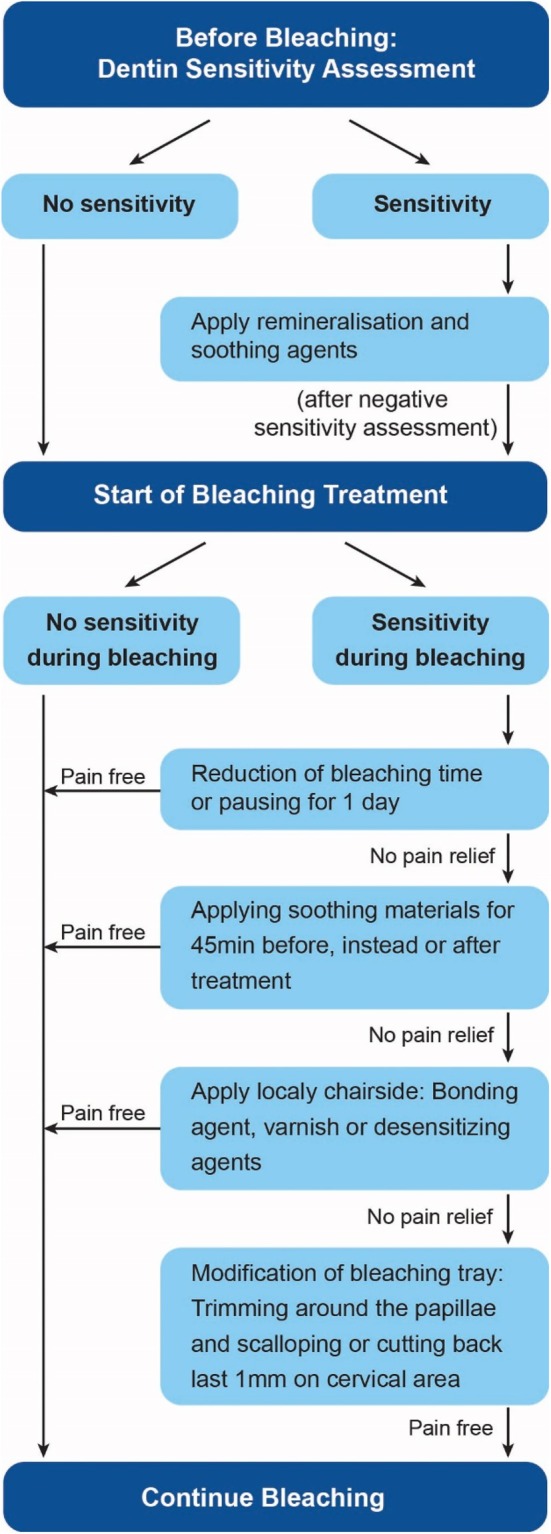
Overview of sensitivity management. Examples of desensitizing and soothing agents include casein phosphor–peptide–amorphous calcium phosphate (CPP‐ACP), potassium nitrate, fluoride, or gluma‐based products.

Before treatment, professional cleaning is recommended to remove surface stains and substances that could interfere with bleaching. Furthermore, it is essential to assess and address dentin hypersensitivity, as many MIH patients have preexisting discomfort due to tooth mottling (see Figure 2).

While various bleaching approaches are available, home‐bleaching is recommended. Unlike in‐office bleaching, which typically uses high‐concentration gels (e.g., 6%–35% hydrogen peroxide), home‐bleaching involves low concentration peroxide gels (e.g., 5%–16% carbamide peroxide gels containing an effective hydrogen peroxide concentration of 1.8%–6%, respectively), thereby minimizing the risk of bleaching sensitivity. This makes it especially suitable for younger patients [14, 15, 34, 35].

Due to the lower concentration, treatment is carried out over several weeks using custom‐fitted trays, typically worn for 1–8 h per day [14]. The gentler nature of home bleaching enables the dentist and patient to gradually determine the optimal result. Progress should be assessed every 2–3 weeks, with treatment considered complete once the desired result is achieved or no further improvement is observed between assessments. In basic cases, a 4–6 week bleaching period might be sufficient, while advanced or complex cases might require 8–12 weeks or longer (Table 1, Figure 1). The yellow–brown discoloration in MIH lesions is often attributed to elevated protein levels incorporated during development, typically located deep within the lesion [36, 37]. Consequently, longer bleaching times are required compared with discolorations closer to the surface. However, the proposed durations should be regarded as general guidelines, as the actual treatment time depends on multiple factors and may vary considerably between cases [22, 30, 38, 39, 40].

While bleaching typically aims to remove yellow or brown discolorations, as illustrated in Figure 1, it may also help to mask white spots by lightening the surrounding enamel. It should be noted that due to dehydration and demineralization, white spots may initially appear more pronounced or mottled after treatment. These changes are temporary and will disappear as the enamel rehydrates and remineralizes [41, 42].

In addition to removing the discolorations and improving OHRQoL [43], bleaching can also improve the oral health of patients by inhibiting plaque formation. It has furthermore been reported that bleaching can promote a reduction in gingival bleeding, which can also be explained by a decrease in plaque accumulation in the gingival margin due to penetration of the bleaching agent [44, 45, 46, 47, 48].

### 2.3 Phase II—Microinvasive Treatment (Resin Infiltration and/or Microabrasion)

Depending on the outcome of home‐bleaching and the complexity of the defect, subsequent microinvasive treatments might be required (Figure 1). In basic cases, discoloration is typically sufficiently removed such that no further intervention is required. However, in more complex cases, residual whitish opacities frequently persist. These are caused by air and water trapped within the porous structure of the lesion and become visible once the darker discoloration has been removed.

To effectively mask these residual opacities and reinforce the porous enamel, resin infiltration is particularly recommended. This procedure should be performed after a minimum waiting period of 2 weeks. This allows for the dissipation of residual peroxides, preventing a reaction with the resin molecules, and ensures stabilization of the tooth shade.

When performing resin infiltration, optimized protocols such as Transillumination‐Aided Infiltration [23] or Infiltration Monitoring by Transillumination [22] are advised. These approaches use transillumination both as a diagnostic aid, to guide surface layer removal necessary for effective infiltration, and as a monitoring tool during the infiltration process. Such approaches have demonstrated high success rates in masking the whitish opacities of the defect [9, 10, 11, 18, 19, 21, 22, 23, 24, 25, 26, 27, 28].

Microabrasion might also be considered as either a complementary step to resin infiltration, that is, to remove the superficial surface layer to enable access to the lesion, or as a standalone treatment for persistent surface opacities that may remain after bleaching [16]. However, since it is a purely abrasive method and MIH lesions are often complex and deep, its use should be approached with caution to avoid unnecessary and excessive enamel loss.

### 2.4 Phase III—Invasive Treatment, If Indicated (Composite Restoration)

In most cases, treatment is complete following microinvasive procedures, as the opacities are sufficiently masked. However, in more complex cases, a significant loss of tooth structure may be present—either due to enamel breakdown, previous trauma, or as a consequence of extensive microinvasive treatment, which might be required to access deep lesions in some cases. In such situations, localized composite restorations are necessary to replace the lost structure.

## 3 Bleaching Sensitivity Management

Although home bleaching uses low‐concentration peroxide, mild and temporary side effects can still occur, with transient bleaching sensitivity being the most common, typically due to reversible pulpitis [14, 49]. Theoretically, children and adolescents may experience greater bleaching sensitivity than adults due to thinner dentin [50]. However, younger patients generally have fewer enamel cracks, less gingival recession, and a pulp with greater regenerative capacity, all of which may help mitigate their perception of bleaching sensitivity [51]. While MIH‐related enamel damage can increase the risk of bleaching sensitivity, this is primarily a concern for MIH‐affected molars and less so for anterior teeth. Nonetheless, careful sensitivity management is essential.

To manage such events, patients should be instructed in appropriate sensitivity management (Figure 2) and should be able to get in contact with their dentist during this period to alleviate any concerns. Ideally, patients should be provided with a sensitivity management sheet to record their bleaching sensitivity daily, for evaluation during follow‐up assessments. Management recommendations may vary depending on the situation; pausing the bleaching procedure and/or applying desensitizing materials—such as casein phosphor–peptide–amorphous calcium phosphate (CPP‐ACP), potassium nitrate, fluoride, or gluma‐based products [52]—are measures that can be recommended (Figure 2).

Another side effect is gingival irritation, caused by direct contact during home bleaching. If this occurs, pausing the bleaching treatment is recommended.

## 4 Clinical Examples

**FIGURE 3:**
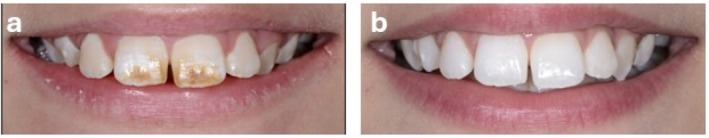
Basic case. (a) Brown, mottled discoloration at baseline. At‐home bleaching (2 weeks 5% and 4 weeks 10% carbamide peroxide) was performed with (b) showing results after 6 weeks.

An 11‐year‐old patient sought treatment due to brown, mottled discoloration on her anterior teeth (Figure 3, baseline). The orthodontic situation, Class II Division 1 malocclusion, made it difficult for the patient to fully close her mouth, promoting enamel dehydration and the accumulation of pigments. The treatment plan included oral hygiene instruction, professional cleaning and at‐home bleaching using customized trays. After an initial 2‐week bleaching phase using 5% carbamide peroxide to minimize the risk of bleaching sensitivity, 10% carbamide peroxide was used for an additional 6–8 weeks in a second bleaching phase. Bleaching was discontinued once the discoloration had resolved (Figure 3, after bleaching). No further treatment was necessary.

**FIGURE 4:**
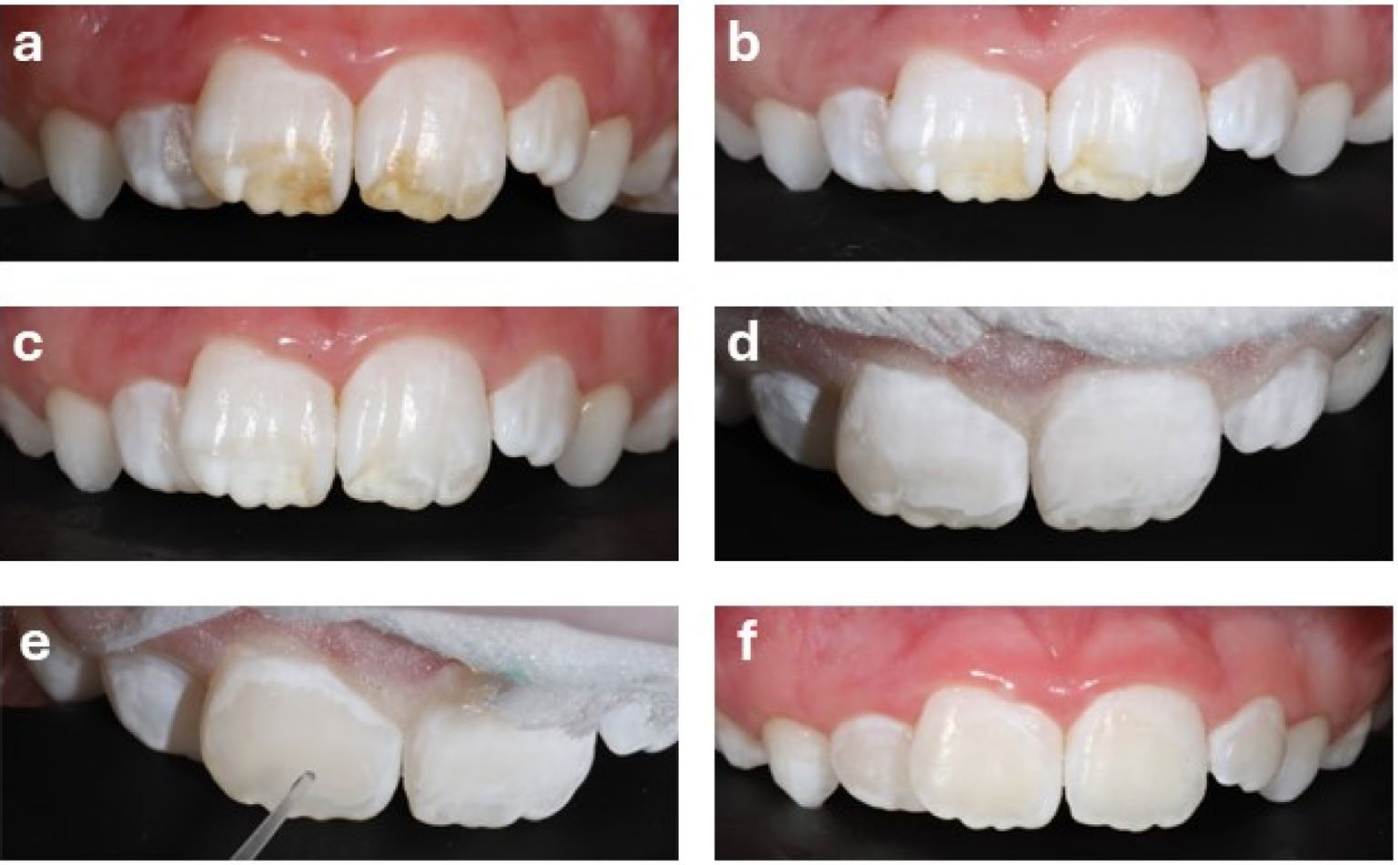
Advanced case. (a) Brown discoloration at baseline. At‐home bleaching (10% carbamide peroxide) was performed with results shown after (b) 2 and (c) 6 weeks. Following a 2 week wash out period resin infiltration was performed to mask residual white opacity. Images show the results after (d) 2 min etching; (e) drying, and (f) infiltration with the resin infiltrant.

A child aged 9, was teased at school due to brown discoloration on his teeth. This negatively affected his confidence and social interactions (Figure 4, baseline). Motivated to improve his wellbeing and quality of life, both he and his mother actively sought treatment. Following professional cleaning, initial treatment involved at‐home bleaching with 10% carbamide peroxide, delivered via custom‐fitted bleaching trays fabricated in‐house using 3D printing. The bleaching treatment was carried out over a period of six weeks, after which the brown markings had completely resolved. However, faint yellow stains and whitish opacities remained (Figure 4, post‐bleaching), necessitating further treatment with resin infiltration after a 2‐week washout period. The standard resin infiltration protocol was used, which involved an initial cycle of two‐minute etching, cleaning and drying, followed by resin infiltration for three minutes and light curing, and then a second one‐minute infiltration step. This approach successfully masked the remaining opacities to the full satisfaction of the patient and his mother (Figure 4, after resin infiltration). No side effects were reported.

## 5 Discussion

Many children seeking treatment are 11–12 years old, transitioning from primary to high school. At this sensitive stage, they are especially self‐conscious and reluctant to enter a new social environment due to visible tooth discoloration. These children often present with mixed dentition and developing malocclusions, most commonly Class II Division 1, with features such as incompetent lips, high lip lines, short lips, and mouth breathing. This results in dehydration of the upper central incisors, which are often already compromised by enamel defects such as MIH, thus making opacities more prominent.

Whereas the whitish appearance of opacities is caused by air trapped within the porous structure of defects, yellow and brown discolorations are linked to markedly higher levels of serum proteins such as albumin—approximately eightfold and 15–21‐fold increases, respectively—suggesting incorporation during the formation of the MIH defect [36]. In addition, structural defects render lesions more susceptible to bacterial infiltration and external pigments, further intensifying discoloration [37]. This effect is exacerbated by poor oral hygiene: porous enamel facilitates the adhesion of tannins and other staining agents, while biofilm accumulation on dehydrated, protruded anterior teeth further traps stains. As a result, the characteristic yellow–brown discolorations are often mistaken for inadequate brushing. Unfortunately, these children are often teased, further lowering self‐esteem and contributing to a cycle of neglect and worsening discoloration.

The primary aim of treatment is thus to remove these opacities and restore a normal appearance. As patients begin to see the results of the treatment, that is, as brown and yellow stains are gradually removed, there is often a noticeable improvement in oral hygiene and self‐esteem. Seeing a cleaner, whiter smile in the mirror motivates better care and confidence.

When developing an individual treatment plan, it is not necessary for it to be fixed from start to finish. The stepwise approach described in this article, beginning with noninvasive techniques and progressing to more advanced interventions as needed based on treatment outcomes, allows for optimal results while preserving as much natural tooth structure as possible, thus supporting oral health and overall wellbeing.

While at‐home bleaching is often effective in eliminating discoloration, residual whitish opacities commonly persist. In such cases, resin infiltration is typically required to mask these opacities and stabilize the porous structure of the lesion. Moreover, at‐home bleaching is also recommended as an initial step for treating whitish demarcated opacities. This helps address deeper discolorations that might otherwise become more visible following resin infiltration, as the reduction in light scattering through the porous enamel allows for a clearer view into the lesion.

The risk of recurrent discoloration in MIH lesions following treatment appears minimal. This can be attributed to the irreversible reaction of peroxides with discoloration‐causing molecules and the protective effect of resin infiltration, which creates an internal barrier thus preventing penetration of external pigments. While new extrinsic discoloration from dietary sources may occur, this risk is comparable to that of conventional restorations and can be effectively managed with subsequent bleaching, as demonstrated for both resin composites and resin‐infiltrated lesions [53, 54]. Although long‐term data specific to MIH cases are limited, current evidence suggests stable outcomes for at least 1–2.5 years following bleaching and 6 years after resin infiltration [55, 56]. These findings support the clinical longevity of conservative approaches while highlighting the importance of ongoing oral hygiene and the availability of retreatment options if needed.

## 6 Conclusion

When treating demarcated anterior opacities, a stepwise approach, starting with noninvasive techniques and advancing to more complex interventions as needed based on treatment outcomes, is recommended. This strategy allows for optimal results while preserving as much natural tooth structure as possible, thus supporting oral health and overall wellbeing. At‐home bleaching is suggested as an initial step in the treatment of demarcated opacities on anterior teeth due to its effective, safe, and noninvasive application.

## Author Contributions

**Linda Greenwall:** conceptualization, clinical procedures, investigation, editing. **Marcus Cebula:** methodology, writing – review and editing, supervision. **Joseph Greenwall‐Cohen:** review and editing. **Falk Schwendicke:** conceptualization, methodology, writing – review and editing, supervision. **Susanne Effenberger:** conceptualization, methodology, writing – original draft.

## Conflicts of Interest

J.G.C. does not have any financial interest in the companies whose materials are included in this article. M.C. and S.E. are employees of DMG Dental‐Material Gesellschaft mbH, the company that is marketing the commercial resin infiltrate Icon, but they do not receive any personal benefits from the sale of this product. L.G. and F.S. are consultants and give lectures for DMG Dental‐Material Gesellschaft mbH but do not receive any personal benefits from the sale of the commercial resin infiltrate Icon. S.E. is a part‐time employee of the department headed by FS.

## Data Availability

The data that support the findings of this study are available on request from the corresponding author. The data are not publicly available due to privacy or ethical restrictions.
